# Clinical Impact of Sagittal Spinopelvic Parameters on Disc Degeneration in Young Adults

**DOI:** 10.1097/MD.0000000000001833

**Published:** 2015-10-23

**Authors:** Young-Min Oh, Jong-Pil Eun

**Affiliations:** From the Department of Neurosurgery, Research Institute of Clinical Medicine of Chonbuk National University-Biomedical Research Institute of Chonbuk National University Hospital, Jeonju, Korea.

## Abstract

The sagittal balance plays an important role in the determination of shear and compressive forces applied on the anterior (vertebral bodies and intervertebral discs) and posterior (facet joints) elements of the lumbar vertebral column. Many studies have also examined the effect of structural changes in the disc on the biomechanical characteristics of the spinal segment. Nevertheless, the relationship between sagittal balance and the degree of disc degeneration has not been extensively explored. Thus, here we investigated the relationships between various sagittal spinopelvic parameters and the degree of disc degeneration in young adults.

A total of 278 young adult male patients were included in this study (age range: 18–24 years old). Multiple sagittal spinopelvic parameters, including pelvic incidence (PI), sacral slope (SS), pelvic tilt (PT), lumbar lordosis (LL), sacral inclination (SI), lumbosacral angle (LSA), and sacral table angle (STA), were measured from standing lateral lumbosacral radiographs. The degree of intervertebral disc degeneration was classified using a modified Pfirrmann scale. To assess the pain intensity of each patient, the visual analogue scale (VAS) score for low back pain (LBP) was obtained from all the patients. Finally, the relationships between these spinopelvic parameters and the degree of disc degeneration in young adults were analyzed. Also, we performed multiple logistic regression study.

Out of all the spinopelvic parameters measured in this study, a low STA and a low SI were the only significant risk factors that were associated with disc degeneration in young adults. It means that patients with disc degeneration tend to have more severe sacral kyphosis and vertical sacrum.

We found that patients with disc degeneration showed a lower SI and lower STA compared with patients without disc degeneration in young adults. Therefore, we suggest that the patients with disc degeneration tend to have more vertical sacrum, more sacral kyphosis, and more severe LBP, and that SI and STA measurements should be carefully considered to predict or prevent further disc degeneration and LBP.

## INTRODUCTION

The sagittal balance plays an important role in the determination of shear and compressive forces applied on the anterior (vertebral bodies and intervertebral discs) and posterior (facet joints) elements of the lumbar vertebral column.^1^ Many studies have also examined the effect of structural changes in the disc on the biomechanical characteristics of the spinal segment.^2–4^ Recently, Keorochana et al^5^ reported that sagittal alignment may alter spinal load and mobility, thereby possibly influencing segmental degeneration; moreover, alterations in sagittal alignment may lead to kinematic changes that influence load bearing and the distribution of disc degeneration at each level.

Nevertheless, the relationship between sagittal balance and the degree of disc degeneration has not been extensively explored. Moreover most of these subjects were old age (more than 40 years old). Because the degree and level of disc degeneration were related to old age, occupation, life style, and body mass index, these factors should be excluded.^6–8^ Thus, here we investigated the relationships between various sagittal spinopelvic parameters and the degree of disc degeneration in young adults.

## METHODS

### Study Population

We performed a retrospective cross-sectional study. From 2009 to 2012, among all of the patients visited our outpatient department and complained of lower back pain, a total of 278 young adult male patients who ranged in age from 18 to 24 years (mean, 20.9 ± 1.7 years), and were our college students, enrolled in this retrospective study. Detailed patient demographic data are shown in Table 1. To minimize confounding factors such as bone remodeling, secondary morphologic changes, age-related differences, and sex-related differences, only male patients aged 18 to 24 years old in our college students were included. Exclusion criteria for this study comprised scoliosis, Scheuermann disease, hip pathology, and indecipherable femoral heads as visualized on lateral lumbosacral radiographs. Institutional Review Board of Chonbuk National University Hospital approved this study.

**TABLE 1:**
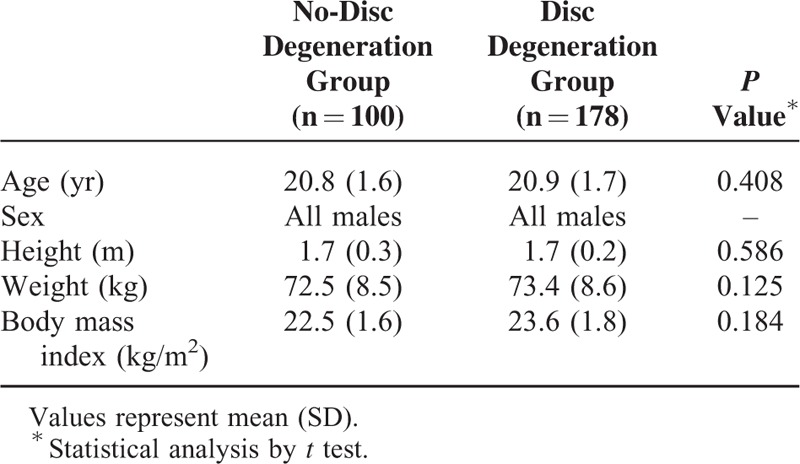
Demographics of the Populations

Standing lateral films that included both hip joints were obtained for each patient. Films were obtained while the patient was placed in a standing position with his arms folded on his chest and his knees fully extended. Pelvic incidence (PI), sacral slope (SS), and pelvic tilt (PT) were measured according to the method described by Legaye et al.^9^ Lumbar lordosis (LL) and sacral inclination (SI) were measured using the method described by Wiltse et al.^10^ Lumbosacral kyphosis was evaluated using the lumbosacral angle (LSA), which is the angle between the superior endplate of L5 and the posterior cortex of S1, as described by Dubousset et al.^11^ To evaluate the tilting angle of the sacral endplate, the sacral table angle (STA) was measured between the line along the sacral endplate and the line drawn along the posterior aspect of the S1 vertebral body, according to the method used by Österman et al.^12^ Since this was a retrospective study, the sacro-femoral distance (SFD) was measured instead of using the C7 plumb line to measure the sagittal vertical axis. The SFD is the horizontal distance between the vertical bi-coxo-femoral axis and the vertical line passing through the posterior corner of the sacrum.^13^ All radiographic measurements were calculated from the existing lateral lumbosacral radiographs and were performed twice by 2 spine surgeons (Y-M O and J-P E) using picture archiving and communication systems (PACS) software. The intraobserver and interobserver variability of these measurements was ±1°, with intraclass correlation coefficients ranging between 0.93 and 0.99.

The degree of intervertebral disc degeneration was classified using a modified Pfirrmann scale, which is an MRI-based assessment.^14^ Patients were divided into 2 groups according to their Pfirrmann grade. The “no-disc degeneration” group (n = 100) included all patients without any disc degeneration (Pfirrmann grade I), whereas the “disc degeneration” group (n = 178) comprised all patients who showed at least some degree of disc degeneration (Pfirrmann grade II to V). Among the “disc degeneration” group, 80 patients showed disc degeneration on L4/5 level, 65 patients on L5/S1 level, and 33 patients showed disc degeneration on both L4/5 and L5/S1 levels.

To assess the pain intensity of each patient, the visual analog scale (VAS) score (0, no pain; 10, worst pain) for low back pain (LBP) were obtained from all the patients.

### Statistical Analysis

The independent samples *t* test was used to compare the VAS score and the spinopelvic parameters of these 2 groups. For multivariate analysis, optimum cut-off values were determined by identifying the highest positive likelihood ratio (sensitivity/[1 − specificity]) among all variables with a *p* value less than 0.1 in the *t* test. Univariate and multivariate logistic regression analyses were used to calculate the odds ratios (ORs) for the risks associated with disk degeneration. All data were analyzed using SPSS 21.0 (IBM SPSS Inc, Chicago, IL); *p* values less than 0.05 were considered statistical significance.

## RESULTS

### Difference of VAS Score Between 2 Groups

A total of 178 patients exhibited disc degeneration (Pfirrmann grade II to V), whereas 100 patients did not exhibit any disc degeneration (Pfirrmann grade I). There was a significant difference in VAS scores between the disc degeneration group and the no-disc degeneration group. The mean VAS scores were 4.8 in the no-disc degeneration group and 6.0 in the disc degeneration group (*P* < 0.001).

### Relationship Between Spinopelvic Parameters and Disc Degeneration

**FIGURE 1:**
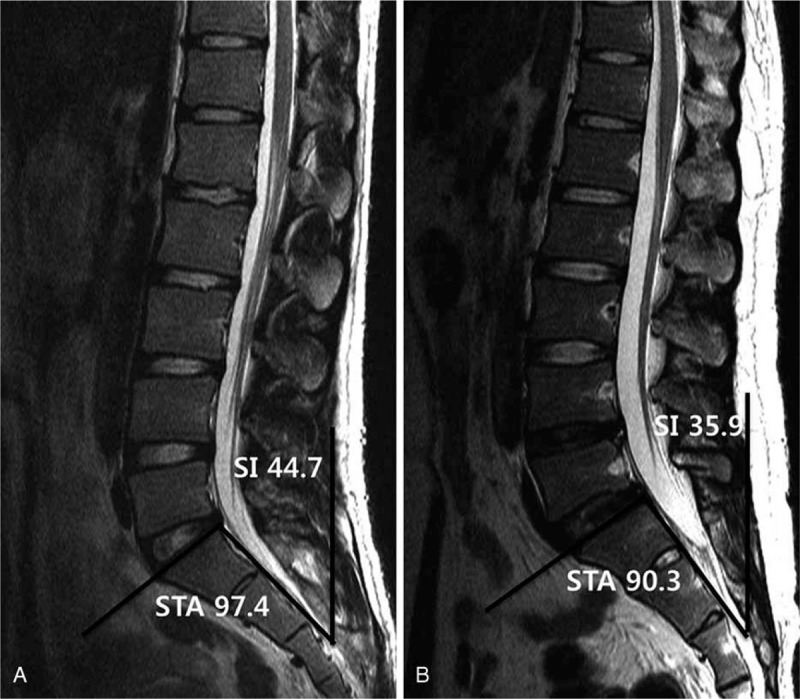
A, It denotes a magnetic resonance image (MRI) of a patient without disc degeneration and (B) shows an MRI of a patient with disc degeneration. The sacral inclination (SI) and sacral table angle (STA) are lower in a patient with disc degeneration (B) than in a patient without disc degeneration (A). The sacrum of the patient with disc degeneration is more vertical and showed more kyphosis (B) than that of the patient without disc degeneration (A).

The LSA and STA values were significantly lower on average in the disc degeneration group compared with the no-disc degeneration group. The mean LSA values were 116.8° ± 6.5 in the no-disc degeneration group and 114.0° ± 7.1 in the disc degeneration group (*P* < 0.001). The mean STA values were 97.4° ± 6.1 in the no-disc degeneration group and 95.4° ± 6.1 in the disc degeneration group (*P* *=* 0.008). An MRI scan of a patient without disc degeneration is shown in Figure 1A, whereas an MRI scan of a patient with disc degeneration is shown in Figure 1B. The extent of LL was higher in the no-disc degeneration group (31.9° ± 10.6) compared with the disc degeneration group (29.8° ± 11.4); however, this difference was not significant (*P* *=* 0.129). Other spinopelvic parameters such as PI, SS, PT, SFD, LL, and SI were also not significantly different between the 2 groups (Table 2). We checked the normality test and homogeneity of variance for independent samples *t*-test assumption and there were all acceptable for *t* test.

**TABLE 2:**
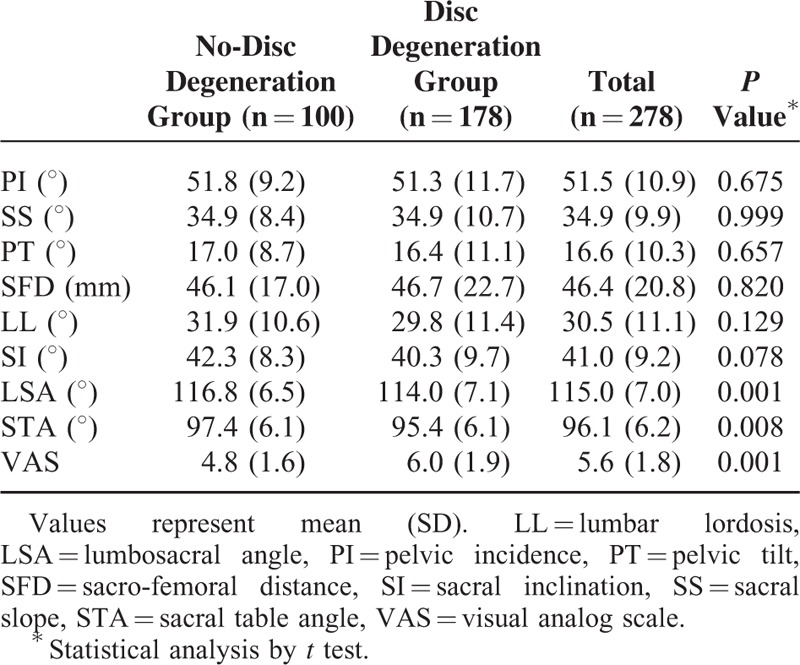
Comparison of the Spinopelvic Parameters and VAS Scores Between Disc Degeneration Group and No-Disc Degeneration Group

### Factors Associated With Disc Degeneration

Although the differences between the 2 groups regarding the SI, LSA, and STA values were not significant according to the independent *t* test, the *P* values of these comparisons were all less than 0.1. Highest positive likelihood ratio analysis identified cut-off values for SI, LSA, and STA of 37°, 114°, and 99.5°, respectively. The ORs of the low SI and low STA values were 1.923 [95% confidence interval (CI) 1.092–3.388] and 2.219 (95% CI 1.300–3.788), respectively (Table 3). However, the LSA was not correlated with disc degeneration according to multiple logistic regression analysis. Therefore, we suggested that a low STA and a low SI are only 2 factors related to the disc degeneration in young adults.

**TABLE 3:**
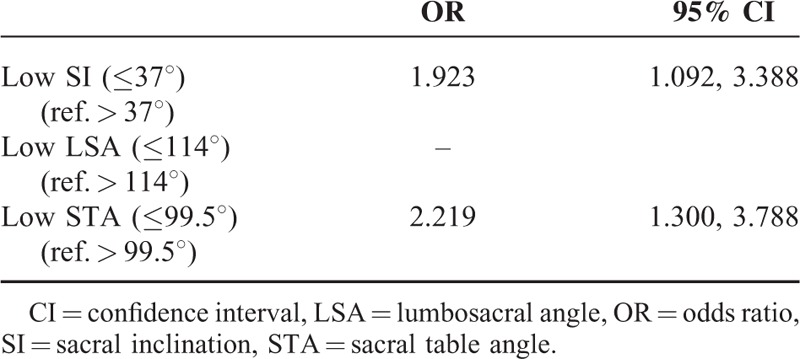
Multiple Logistic Regression Analysis for the Risk Factors Associated With Disc Degeneration Among Young Adults Male

## DISCUSSION

Several other studies have investigated the associations between various spinopelvic parameters and lumbar disc herniations.^15–20^ Rajnics et al and Endo et al^16,17^ reported a common pattern of spinopelvic sagittal alignment in patients with lumbar disc herniations, which was characterized by a low SS, a low LL, and an anterior translation of the C7 plumb line. Barry et al^13^ concluded that PI, SS, and LL values are all lower in patients with lumbar disc herniations. Recently, Yang et al^20^ found that patients with lumbar disc degenerative disease had a significantly lower PI as well as a more vertical sacrum, a flatter spine, and a more anterior translation of the C7 plumb line compared with normal subjects. The authors proposed that a lower PI would result in a lower SS and PT, which would then lead to a flatter LL and thoracic kyphosis (TK); therefore, PI may play a predisposing role in the pathogenesis of lumbar degenerative disease. However, these studies were limited in that the mean age of the subjects was greater than 40 years. Because the degree and level of disc degeneration were also probably related to old age, occupation, and overweight status, all of these factors are potentially confounding and would ideally have been excluded. In addition, these studies did not measure the STA, SI, or LSA.^6–8^

We included only young adults aged 18 to 24 to minimize confounding factors of disc degeneration such as age, obesity status, body mass index (BMI), and occupation. Among those in the disc degeneration group in our study, 80 patients showed disc degeneration at L4/5 level, 65 patients at L5/S1 level, and 33 patients showed disc degeneration at both L4/5 and L5/S1 levels.

In this study, the VAS scores were significantly higher on average in the disc degeneration group compared with the no-disc degeneration group. These results show that the patient with disc degeneration have more severe LBP than the patient without disc degeneration. And the degree of disc degeneration is related to the spinopelvic parameters such as SI and STA. Therefore, we posit that LBP is affected by the degree of disc degeneration as well as the spinopelvic parameters such as SI and STA.

According to the independent samples *t* test, the LSA and STA were significantly different between the disc degeneration group and the no-disc degeneration group (Table 2). However, we did not observe any significant differences in the PI, SS, PT or LL values between the 2 groups, unlike previous studies.^13,16,17^ Previous studies reported that compensatory pelvic retroversion in patients with degenerative disc disease might induce decreased SS and increased PT, because the aging spine was not flexible. However, according to our study, we think that there is a compensatory hyperextension in young adult patients with back pain, because the spine of young adults is still flexible.

Since confounding effects among the factors investigated here may have affected our results, we determined the SI, LSA, and STA cut-off values and performed multiple logistic regression analysis using these possible risk factors. This analysis revealed that SI and STA were the only significant risk factors associated with disc degeneration (Table 3). The OR of disc degeneration in patients with a SI ≤ 37° was 1.923 (95% CI 1.092–3.388), compared with patients with an SI >37°. Also, the risk of disc degeneration in patients with an STA ≤99.5° was 2.219 (95% CI 1.300–3.788), compared with patients with a STA >99.5°.

STA is an anatomic parameter that represents both sacral morphology and the degree of sacral kyphosis. Thus, a lower STA indicates that the extent of sacral kyphosis is more severe. In this study, the average STA was lower in the disc degeneration group than in the no-disc degeneration group. Therefore, we suggest that sacral kyphosis aggravates disc degeneration. Previously, Wang et al^21^ reported that a low STA, which translates into a steeper SS with a higher shear stress, could predispose patients to further forward slippage; thus, this study concluded that STA is an important factor in the etiology of L5-S1 spondylolisthesis in both children and adolescents. Furthermore, Whitesides et al^22^ found that a low STA indicates a higher shear stress on the disc. Therefore, we suggest that a low STA is related to disc degeneration.

In 2012, Tanguay et al^23^ investigated the clinical significance of the LSA, and concluded that increased lumbosacral kyphosis has a significant association with a decrease in the physical aspect of the quality of life for patients with adolescent L5-S1 spondylolisthesis. Recently, Oh et al^19^ studied the relationship between spinopelvic parameters and disc degeneration in patients with spondylolysis, and concluded that a lower LSA aggravates disc degeneration. In our study, the LSAs of the 2 groups were significantly different according to the *t* test; however, the LSA was not correlated with disc degeneration according to multiple logistic regression analysis. Since the LSA is closely related to the STA (Pearson's correlation coefficient 0.485, *P* < 0.001; data not shown), we hypothesize that LSA could be a confounding factor for disc degeneration.

The SI describes the relationship of the sagittal plane of the sacrum with the vertical plane. Normally when the patient stands the sacrum is inclined forward. In this study, a low SI was a significant risk factor for disc degeneration. A low SI means that the presentation of the sacrum is more vertical. Therefore, a more vertical sacrum could also be a risk factor for disc degeneration. Previously, Wiltse et al^10^ reported that the sacrum tends to become more vertical with increasing degrees of spondylolisthesis, and that the angle of inclination becomes correspondingly smaller.

The sacrum of a representative patient with disc degeneration is more vertical and shows more kyphosis (Fig. 1B) than that of a representative patient without disc degeneration (Fig. 1A). Thus, if the sacrum is more vertical and shows more kyphosis, the patient has a lower SI and STA. Therefore, we suggest that a more vertical sacrum and a more severe sacral kyphosis are associated with disc degeneration. Based on our results, we think that the efforts to reduce vertical sacrum and sacral kyphosis, such as extension-traction method, would prevent further disc degeneration.

Since this study is retrospective and cross-sectional, it has some limitations. For example, occupation, sitting position, and exercise are some of the other possible risk factors that we did not evaluate in this study. Second, this is a cross-sectional study, so it is not clear whether the relationships between disc degeneration and spinopelvic parameters are causal or merely correlative. Further comprehensive prospective studies are needed to answer these questions.

## CONCLUSIONS

We found that patients with disc degeneration showed a lower SI, lower STA, and more severe LBP compared with patients without disc degeneration in young adults. Therefore, we suggest that the patients with disc degeneration tend to have more vertical sacrum, more sacral kyphosis, and more severe LBP, and that SI and STA measurements should be carefully considered to predict or prevent further disc degeneration and LBP.
